# Protein Biomarkers in Glaucoma: A Review

**DOI:** 10.3390/jcm10225388

**Published:** 2021-11-18

**Authors:** Ewa Fiedorowicz, Anna Cieślińska, Patrycja Kuklo, Andrzej Grzybowski

**Affiliations:** 1 Department of Biochemistry, Faculty of Biology and Biotechnology, University of Warmia and Mazury, Oczapowskiego 1A Street, 10-719 Olsztyn, Poland; ewa.kuzbida@uwm.edu.pl (E.F.); anna.cieslinska@uwm.edu.pl (A.C.); 2 Department of Ophthalmology, University of Warmia and Mazury, 10-719 Olsztyn, Poland; patrycja.kuklo@uwm.edu.pl; 3 Institute for Research in Ophthalmology, Foundation for Ophthalmology Development, 61-553 Poznan, Poland

**Keywords:** glaucoma, biomarker, peptide, protein, glaucoma screening, eye-disorder screening

## Abstract

Glaucoma is a multifactorial disease. Early diagnosis of this disease can support treatment and reduce the effects of pathophysiological processes. A significant problem in the diagnosis of glaucoma is limited access to the tested material. Therefore, intensive research is underway to develop biomarkers for fast, noninvasive, and reliable testing. Biomarkers indicated in the formation of glaucoma include chemical compounds from different chemical groups, such as proteins, sugars, and lipids. This review summarizes our knowledge about protein and/or their protein-like derived biomarkers used for glaucoma diagnosis since 2000. The described possibilities resulting from a biomarker search may contribute to identifying a group of compounds strongly correlated with glaucoma development. Such a find would be of great importance in the diagnosis and treatment of this disorder, as current screening techniques have low sensitivity and are unable to diagnose early primary open-angle glaucoma.

## 1. Introduction

Glaucoma refers to a group of optic neuropathies with characteristic morphological changes in the retinal nerve fiber layer and the optic nerve head (ONH). These changes are associated with slow and progressive retinal ganglion cell (RGC) death, characteristic changes in neuroretinal rim tissue in the ONH, and visual field loss [1,2]. Primary open-angle glaucoma (POAG) is the most prevalent form of glaucoma in the Western world [3,4,5].

**Figure 1:**
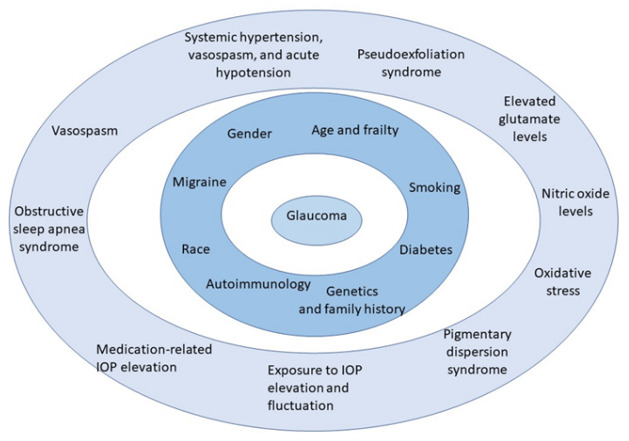
Risk factors influencing glaucoma development.

Glaucoma is a multifactorial disease that may be correlated with immune reaction, ischemia, and oxidative stress [6,7,8,9,10,11]. The most important risk factors of disease development are shown in Figure 1 [2,12,13,14,15,16].

One of the most important problems facing the field of ophthalmology is determining how to diagnose glaucoma early. So far, the threat of blindness is prevented by timely treatment through the lowering of intraocular pressure (IOP). The diagnosis of glaucoma requires a detailed examination of the optic disc structure and visual field; combinations of patient history and objective methods for the evaluation of the ONH, including the retinal nerve fiber layer (RNFL), visual fields, tonometry, and corneal thickness; and assessing the structure and function of the eye. Potential screening tests classify subjects as healthy, as glaucoma suspects, or as having glaucomatous pathology of an insufficient predictive power [17,18]. A significant problem in diagnosing the disease is limited access to the tested material. The process of neurodegeneration occurs in the optic nerve and RGCs; examination of these tissues in patients is not feasible. Less invasive and more accessible clinical testing for glaucoma could be improved if specific biomarkers were detected in body fluids such as the tear film, urine, and whole blood or serum [19,20].

According to the National Institutes of Health’s Biomarkers Definitions Working Group, a biomarker is defined as a characteristic that is objectively measured and evaluated as an indicator of normal biological processes, pathogenic processes, or pharmacologic responses to therapeutic intervention and has valuable applications in disease detection and monitoring of health status [21]. To identify a biomarker for clinical utility, it must be confirmed as valid, reproducible, specific, and sensitive. Biomarkers are needed for early diagnosis of this blinding disease, and prediction of its prognosis could promote precise treatment [22]. One of the important challenges of serum biomarker detection is related to the much lower abundance of most proteomic biomarkers than some disease-irrelevant serum proteins [23].

Among the biomarkers indicated in glaucoma formation are chemical compounds belonging to different chemical groups such as proteins, sugars, and lipids. In this review, we systematize the knowledge gained since 2000 about biomarkers characteristic for glaucoma, and we provide an overview of biomarkers in biochemical groups on protein and/or their protein-like derivation. Herein, we also include suggestions for appropriate research methods that will allow their detection in the biological material of patients.

## 2. Proteins

A protein comprises one or more long chains of amino acid residues. Because of their various biological functions, proteins can be categorized as enzyme catalysts, structural proteins, hormones, transfer proteins, antibodies, storage proteins, and protective proteins [24]. Selected proteins shown to be correlated with glaucoma development are presented in Table 1.

Farkas et al. [38] showed that elevated ferritin, an iron-regulating protein, is present in glaucoma. Serum ferritin is related to oxidative stress and inflammation. Lin et al. [35] revealed that an increased serum ferritin level was associated with a greater probability of glaucoma in a representative sample of South Koreans, and an increased serum ferritin level was associated with a high risk of glaucoma in men but not in women [34].

Nerve growth factor (NGF) and brain-derived neurotrophic factor (BDNF), members of the neurotrophin family, have been shown to control a number of aspects of survival, development, and function of neurons in both the central and peripheral nervous systems [39,40,41]. Several studies indicate that NGF and BDNF are involved in RGC survival [29]. Ghaffariyeh et al. [30,31] suggested that BDNF found in tears might be a useful biochemical marker for early detection of normal-tension glaucoma (NTG). They proposed that identification of this biomarker might be a reliable, time-efficient, and cost-effective method for diagnosing, screening, and assessing the progression of POAG. Oddone et al. [29] showed that BDNF and NGF serum levels were reduced in the early and moderate glaucoma stages, suggesting the possibility that both factors could be further investigated as potential circulating biomarkers for the early detection of glaucoma.

Wang et al. [37] reported significantly elevated levels of secreted proteins such as cysteine (SPARC), thrombospondin-2, and osteopontin in patients with acute primary angle closure (APAC) compared to the cataract group (*p* < 0.001, *p* < 0.001, and *p* = 0.009, respectively). All four matricellular proteins were found to have a positive correlation with IOP in the current APAC group, but no correlation was found in the previous APAC or cataract groups.

Alterations in sera proteins between patients with POAG, pseudoexfoliation glaucoma (PEXG), and healthy controls were presented by González-Iglesias et al. [36]. The authors identified the 17 most differentially altered proteins overexpressed in the intact serum of newly recruited glaucoma patients. They then proposed a panel of candidates for glaucoma biomarkers and suggested that those candidates are part of a network linked to regulating immune- and inflammatory-related processes.

## 3. Peptides and Amino Acids

Amino acids are the components that serve as substrates for protein synthesis (protein amino acids); they may be referred to as nonprotein amino acids [42]. The studies described here indicate the utility of selected amino acids as biomarkers for glaucoma.

Homocysteine (Hcy) is an amino acid that serves as an intermediate in methionine metabolism to cysteine (Cys) [43]. Researchers proposed a correlation with glaucoma based on studies about the increased risk of cardiovascular diseases [44]. Lin et al. [25] suggested that increased levels of Hcy and Cys may be associated with glaucoma, especially in POAG. However, it may not be useful as a reliable biomarker in glaucoma. In a study from Lopez-Riquelme et al. [28], Hcy levels were significantly higher (*p* = 0.002) in the POAG group compared to the NTG and control groups. Lee et al. [26] also showed that the Hcy level is associated with the presence of glaucomatous RNFL defects. Conversely, Leibovitzh and Cohen [27] presented a retrospective cross-sectional analysis of the relationship between Hcy and IOP and concluded that Hcy may not be useful as a predictive parameter to recognize subjects prone to the development of elevated IOP. No clinical correlation between the Hcy level and IOP was found.

Two dimethylated isomeric derivatives of the amino acid l-arginine—asymmetric dimethylarginine (ADMA) and symmetric dimethylarginine (SDMA)—were shown to be correlated with advanced glaucoma. The derivative ADMA is an endogenous inhibitor of nitric oxide synthase (NOS), while SDMA is a competitive inhibitor of the cellular uptake of l-arginine, the substrate for NOS. According to the nitric-oxide pathway in glaucoma pathogenesis, these metabolites are associated with endothelial dysfunction [33].

Endothelin-1, a peptide hormone that plays multiple complex roles in the cardiovascular, neural, pulmonary, reproductive, and renal systems [45], was shown to be elevated in the POAG group compared to NTG and control group serum samples [28].

The N-terminal fragment of the proatrial natriuretic peptide (NT-proANP, 1-98) is connected with cardiovascular effects, including the regulation of vascular tone, renal sodium handling, and myocardial hypertrophy. It is synthesized within the heart in response to myocardial stretch, and the development of glaucoma was identified to be associated with elevated levels in the plasma and the aqueous humor of patients with POAG [32]. Peptides and amino acids evaluated as potential biomarkers in glaucoma are presented in Table 1.

## 4. Autoantibodies and Antibodies

Autoantibodies may cause pathology by many different mechanisms and induce disease through a multitude of pathophysiological pathways. These include mimicking receptor stimulation, blocking neural transmission, induction of altered signaling, triggering uncontrolled microthrombosis, cell lysis, neutrophil activation, and induction of inflammation. Within diseases, multiple mechanisms may contribute to clinical manifestation [46].

According to Grus et al. [47], complex antibody profiles are very stable in glaucoma patients. Moreover, it has been suggested that autoantibody profiles (in body fluids such as serum, aqueous humor, or tears) may become powerful and highly specific tools to designate as markers in the diagnosis of glaucoma, characterized by early detection before the appearance of any clinical signs [48]. Gramlich et al. [48] presented a wide range of autoantibodies—such as anti-HSP70, antiphosphatidylserine, g-enolase, glycosaminoglycans, neuron-specific enolase, glutathione-S-transferase, a-fodrin, vimentin, myelin basic protein (MBP), glial fibrillary acidic protein (GFAP), retinaldehyde binding protein, and retinal S-antigen—and their role in glaucoma.

Beutgen et al. [49] showed that phosphoglycerate mutase 1 (PGAM1) levels were significantly different between control and POAG patients. Other autoantigens tested, including ATP synthase subunit alpha (ATP5A1), caldesmon (CALD1), voltage-dependent anion-selective channel protein 2 (VDAC2), and L-lactate dehydrogenase A (LDHA) were not increased.

Using an experimental autoimmune glaucoma (EAG) animal model, Hohenstein-Blaul et al. [18] demonstrated an IOP-independent loss of RGCs, accompanied by antibody depositions and increased levels of microglia. The correlation between neuronal damage and changes in autoantibody reactivity suggests that autoantibody profiling could be a useful glaucoma biomarker. The authors concluded that the absence of some autoantibodies in glaucoma patients reflects a loss of the protective potential of natural autoimmunity and may thus encourage neurodegenerative processes. Furthermore, a number of serum proteins identified by chromatography analysis of human glaucoma may represent diseased tissue-related antigens and serve as candidate biomarkers of glaucoma. However, it is unclear whether the IgG-bound serum proteins identified in this study reflect disease-causing antigens [23]. Joachim et al. [50] compared the entire IgG autoantibody patterns against different ocular antigens (retina, optic nerve, and optic nerve head) in the sera of glaucoma patients and healthy subjects. All groups showed different and complex antibody patterns against the three ocular tissues. Joachim et al. [51] showed the significant differences between the IgG antibody profiles against retinal antigens of the glaucoma groups (PEX and POAG) and controls (up- and downregulations), and the identified biomarkers included heat shock protein 27, α-enolase, actin, and glyceraldehyde-3-phosphate dehydrogenase (GAPDH). Additionally, very complex IgG antibody patterns against retinal antigens were found in all analyzed aqueous humor samples of the NTG and control groups (*p* < 0.001) [51,52].

The findings of Schmelter et al. [53] indicate that glaucoma is accompanied by systemic effects on antibody production and B cell maturation, possibly offering new prospects for future diagnostic or therapy purposes. In total, 75 peptides of the variable IgG domain showed significant glaucoma-related changes. Six peptides were highly abundant in POAG sera, whereas 69 peptides were minimal in comparison to the control group. Table 2 presents autoantibodies and antibodies evaluated as potential biomarkers in glaucoma.

## 5. Cytokines and Growth Factors

Cytokines and growth factors play an essential role in the functioning of the human body, modulating (among others) the immune and nervous systems. They are involved in intercellular communication and transmit signals to the appropriate cells by acting on receptors placed in their cell membranes. High levels of proinflammatory cytokines have been shown to have a significant impact on the development of glaucoma. Li et al. [54] demonstrated that the factor contributing to glaucoma development was the menopausal decrease in hormones in women, with a simultaneous high concentration of proinflammatory cytokine as interleukin-8 (IL-8) in the serum. This finding emphasizes the role of the immune system in the development of glaucoma [6].

Gupta et al. [55] analyzed tear films collected from patients without and with newly diagnosed POAG to assess the concentration of 10 proinflammatory cytokines—IFNγ, IL-10, IL-12p70, IL-13, IL-1β, IL-2, IL-4, IL-6, IL-8, and TNFα. Mean concentrations of tear film cytokines were shown to be lower in the glaucoma group for most of the tested cytokines, among which IL-12p70 may be the most important for diagnosis. The authors concluded that despite the small amount of protein available in the samples, the assessment of tear-film cytokines can be used as an indicator of early POAG.

The remaining cytokines are also considered as factors that may enable the evaluation of glaucoma development. Tumor necrosis factor alpha (TNF-α) has been proven to be a proinflammatory cytokine that can play a role in glaucomatous neurodegeneration. Paschalis [56] showed that increasing concentrations of TNF-α and its receptors (TNFR1 and TNFR2), observed after ocular injury, can contribute to progressive damage to the retina and subsequent glaucoma. This relationship was confirmed even with well-controlled IOP in patients with the Boston Keratoprosthesis (KPro). Similar conclusions were described by Kondkar [57], who observed that an elevated level of tumor necrosis factor alpha (TNF-α) can induce RGC apoptosis and plays a key role in glaucoma neurodegeneration. This relationship was also confirmed in a different group of patients when researchers moderated a positive and significant correlation between the TNF-α level and cup/disc ratio as an important clinical index for pseudoexfoliation glaucoma (PEG) [58]. Therefore, these authors emphasized the possibility of using TNF-α as a biomarker in the early diagnosis of glaucoma and assessment of the severity of the disease.

Growth factors activate the repair mechanisms in human cells by stimulating cells to divide, differentiate, and grow. Serum levels of nerve growth factor (NGF) and BDNF in patients affected by POAG with a wide spectrum of disease severity have proved to be significantly reduced compared to healthy controls [29]. The BDNF influences survival and growth of neurons, serves as a modulator of neurotransmitters, and participates in neuronal plasticity. Its decreased concentration and neurodegenerative effects are observed not only among patients with glaucoma but also those with Parkinson’s and Alzheimer’s diseases [59]. The important actions of insulin-like growth factor-1 (IGF-1) include neurogenesis, angiogenesis, protection against cells in the brain, anti-inflammatory effects, and anti-apoptotic effects. Its serum concentration decreases with increasing age in the elderly population [60,61]. The role of IGF-1 in neurodegenerative diseases is being studied intensively as a factor correlated with defective brain insulin signaling [62]. Dogan et al. [63] showed that IGF-1 levels in serum did not differ in the presence of PEX syndrome, with or without glaucoma.

It is worth emphasizing that PEX is the most common identifiable cause of glaucoma, and aging is the major risk factor. This allows for not just possibilities in the search for glaucoma biomarkers but also those characteristics for neurodegenerative symptoms observed in elderly patients [64,65].

The role of transforming growth factor- β (TGF-β) is to control proliferation and differentiation in most cell types and to act as an anti-inflammatory agent. The frizzled secretion protein (SFRP) family consists of five human-secreted glycoproteins (SFRP1, SFRP2, SFRP3, SFRP4, SFRP5) that play roles in cell signaling. In addition, SFRP1 and SFRP5 may be involved in determining the polarity of photoreceptor cells in the retina.

Guo et al. [66] evaluated bioactive transforming growth factor-β2 (TGFβ2) and secreted frizzled-related protein-1 (SFRP1) levels in the aqueous humor of different types of glaucoma: POAG, chronic angle-closure glaucoma (CACG), primary angle-closure suspects (PACS), and acute angle-closure glaucoma (AACG). The study was performed by means of an ELISA test, and patients with cataracts were considered as a control group. The concentration of this growth factor was significantly higher in the aqueous humor collected from POAG patients compared to control patients. However, this correlation was not identified in CACG, PACS, or AACG patients. Authors have also observed differences in the level of TGFβ2 depending on high and normal IOP in patients from the AACG group. There were no significant differences in the levels of SFRP1 analyzed in aqueous humor collected from tested groups. However, patients with primary POAG with high IOP had lower levels of SFRP1 than patients with normal IOP [66]. The studies assessing the role of cytokines and growth factors in glaucoma are presented in Table 3.

## 6. Hormones and Enzymes

Retinal ganglion cells are known to express estrogen receptors. Prior studies have suggested an association between postmenopausal hormone (PMH) use and decreased IOP, suggesting that sex hormones may play a role in the development of glaucoma and decrease the risk for POAG [67]. Li et al. [68] showed that a decreased level of 17-β-estradiol (E2) in the serum of postmenopausal women is correlated with an increased risk of glaucoma progression. This is consistent with the results of a previous study [67] that confirmed the use of PMH preparations containing estrogens can help reduce the risk of POAG. The proposed mechanism still needs to be confirmed, but it is known that RGCs express estrogen receptors, suggesting that PMH use may reduce the risk of glaucoma development.

Higher levels of adrenocorticotropic hormone (ACTH) were also associated with thinner RNFL globally (*p* = 0.009) and at the inferotemporal (*p* = 0.015), superotemporal (*p* = 0.044), and temporal sectors (*p* = 0.046). Lower adrenal sensitivity was associated with thinner RNFL inferotemporally (*p* < 0.001) and temporally (*p* = 0.037), whereas cortisol level was not associated with RNFL thickness [68].

Canizales et al. [69] analyzed the possibilities of using factors related to oxidative stress as biomarkers for the early diagnosis of glaucoma. The mRNA expression level of several biomarkers of oxidative stress in the aqueous humor was assessed in patients with POAG compared to the control group. Authors have proved that the mRNA expression level of superoxide dismutase 1 (SOD1) is significantly reduced in patients with POAG than in control subjects. Li et al. [70] also studied the relationship between oxidative stress biomarkers, including serum superoxide dismutase (SOD), total antioxidant state (TAS), hydrogen peroxide (H_2_O_2_), malondialdehyde (MDA), glutathione peroxidase, glutathione reductase, and visual field progression in patients with PACG. Serum SOD and TAS levels in the PACG group were significantly lower with simultaneously higher levels of MDA and H_2_O_2_ compared to the control group. These results may indicate that oxidative stress is one of the key factors involved in the formation and development of PACG. The important studies assessing the role of hormones and enzymes in glaucoma are presented in Table 4.

## 7. Uric Acid: An Important Biomarker Combined with Protein Metabolism

Uric acid (UA) is the final product of nitrogen metabolism in humans. Based on its protective effect against oxidative damage [71] in the central nervous system, UA was proposed as a biomarker for POAG. The relationship between serum UA concentration and glaucoma severity was explored. The level of serum UA in the POAG group was approximately 13% lower (*p* < 0.001) than that of the control group. The UA/creatinine (Cr) ratio was approximately 15% lower (*p* < 0.001) in patients with POAG compared with the control group [72]. In addition, the levels of UA were significantly lower in PACG patients compared with control subjects, which makes it an important candidate in reaction to oxidative stress in glaucoma pathogenesis [73]. The studies assessing the role of uric acid in glaucoma are presented in Table 5.

## 8. Detection Methods

Proteomics for the discovery of biomarkers might reveal many important issues, including the inherent differences between biological fluids (and how these differences affect current analytical approaches) and experimental design to maximize efficiency [74]. The method allows us to unravel the biological complexity encoded by the genome at the protein level and is built on technologies that analyze large numbers of proteins in a single experiment [75].

Alterations in sera proteins between patients may be identified through a proven approach utilizing equalization of high-abundance serum proteins with ProteoMiner™ (Bio-Rad, California, USA), two-dimensional fluorescent difference gel electrophoresis (2D-DIGE), matrix-assisted laser desorption/ionization time-of-flight/time-of-flight (MALDI-TOF/TOF), and nanoscale liquid chromatography coupled to tandem mass spectrometry (nanoLC-MS-MS) analysis [36]. A large number of plasma proteins were also observed in tear fluid. The proteins found in tears play an important role in maintaining the ocular surface; changes in tear protein components may reflect changes in the health of the ocular surface. Using reverse-phase high-pressure liquid chromatography (RP-HPLC) and nanoscale liquid chromatography coupled to tandem electrospray ionization mass spectrometry (nanoLC-nano-ESI-MS/MS), 60 tear proteins were identified with high confidence, including well-known abundant tear proteins and tear-specific proteins such as lacritin and proline-rich proteins [76,77]. The search for markers concerns not only peptides and proteins but other groups of chemical compounds as well. Pan et al. [78] conducted a metabolomic analysis of aqueous humor samples using nontargeted gas chromatography combined with a time-of-flight mass spectrometer in patients with POAG undergoing surgery and their results of the patients undergoing cataract surgery. The mean age of the study participants was more than 70 years. The authors identified differences in the metabolomic profiles of the samples obtained from both groups of patients. Reduction of biotin, glucose-1-phosphate, methylmalonic acid, N-cyclohexylformamide 1, sorbitol, and spermidine was observed in POAG patients compared to control subjects. Conversely, it was found that mercaptoethanesulfonic acid 2, D-erythronolactone 2, D-thalose 1, dehydroascorbic acid 2, galactose 1, mannose 1, pelargonic acid, and ribitol were increased in participants with POAG compared to patients with cataracts. The obtained results may contribute to the development of a new therapeutic approach [78].

An example of a technique supporting protein identification is 2D electrophoresis, which allows for the possibility of analyzing proteome profiles to search for protein changes in the levels of pre-existing proteins, induction of new products, or coregulated polypeptides. However, 2D electrophoresis’ limitations include the heterogeneity of biopsy material, the lack of procedures for quantifying protein changes, and the need for better image-analysis systems for supporting gel comparisons and databasing [79].

Methods of antibody-profile detection may be validated with specific antigen microarrays [80], or immunological tests based on antibody responses that could be used for diagnosis and screening purposes [18], or serological proteome analysis (SERPA) for initial autoantibody profiling [49]. The standard techniques also include serological methods such as enzyme-linked immunosorbent assay (ELISA), which offers specific detection of a wide variety of target analytes in different kinds of samples. However, ELISA assays have numerous limitations, such as laborious procedures, the need for a relatively large sample volume, and an insufficient level of sensitivity [81].

Multiple approaches of proteomic technologies are required to cover most of the metabolites. Consequently, metabolome profiling is hampered mainly by its diversity, variation of metabolite concentration by several orders of magnitude, and biological data interpretation [82].

## 9. Conclusions

Currently, there is tremendous interest in research into biological markers in both life sciences and clinical sciences. A biomarker can be used as an unbiased differential indicator of disease onset, as an aid in classifying a disease state, or as an assessment of the severity and progression of the disease. Diagnostic and prognostic biomarkers may be critically useful for the timely treatment of many diseases. Therefore, the search for specific biomarkers is still a challenge and a goal of many clinical and research centers. Intensive research is currently underway to develop biomarkers for the diagnosis of glaucoma.

**Figure 2:**
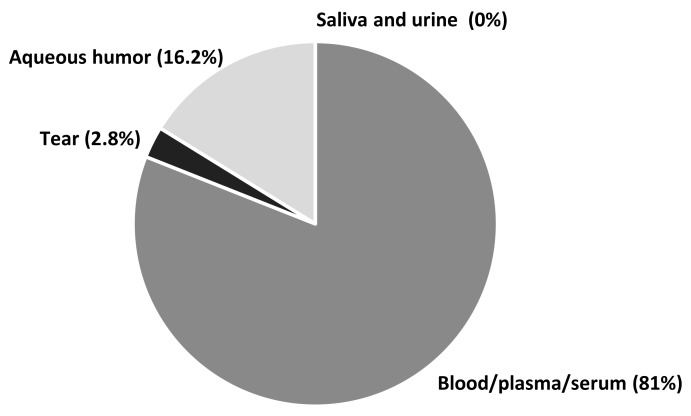
Types and frequency of biological material used for the analysis of potential glaucoma biomarkers.

In relation to the studies presented in this manuscript, most biomarkers were analyzed from blood (serum/plasma) samples. Interestingly, the next biological material used in the analyzed studies was the aqueous humor, although its collection is both difficult and invasive. There were only a few biomarker studies using tears or urine—materials relatively easy to collect. No research was found that tracked protein-biomarkers in saliva (Figure 2).

Limited screening methods and the increasing number of glaucoma patients underscore the need for new biomarkers of POAG. Available clinical analysis tools for glaucoma have limitations, and most glaucoma patients show minimal symptoms at the time of diagnosis. Despite no gold standard for detection of progression, there are available standard automated perimetry, and more recently, optical coherence tomography, as established tests for this purpose. Nevertheless, finding molecular biomarkers and diagnostic factors is imperative in order to predict the occurrence of the disease and develop new treatments. The described possibilities of searching for biomarkers may contribute to the identification of a group of compounds strongly correlated with the development of glaucoma. This is of great importance in diagnosing and treating this disorder, as current screening techniques have low sensitivity and are unable to diagnose early POAG.

## Figures and Tables

**Table 1:** Proteins evaluated as potential biomarkers in glaucoma.

Biomarker	Patient Diagnosis	Number of Patients (*n*)/Biological Material	Results	References
Homocysteine (Hcy)	Primary open-angle glaucoma (POAG), normal tension glaucoma (NTG), ocular hypertension (OHT)	*n* = 44 POAG, *n* = 20 NTG, *n* = 52 OHT, *n* = 78 controls/plasma	Increased levels of Hcy may be associated with glaucoma, especially in patients with POAG. Hcy levels in patients with POAG and NTG groups were significantly higher (*p* = 0.007–0.043).	[25]
Hcy	Glaucomatous retinal nerve fiber layer (RNFL) defect	*n* = 78,049/plasma	The mean Hcy level in the male group with RNFL defects (11.05 ± 3.80 µmol/L) was higher than those without RNFL defects (10.81 ± 4.12 µmol/L (*p* = 0.000, χ^2^ test).	[26]
Hcy	Glaucoma	*n* = 11,850/a retrospective cross-sectional analysis of a database	Mean Hcy levels in subjects with normal intraocular pressure (IOP) of ≤21 mmHg was 11.7 ± 5.5 μmol/L and 12.09 ± 3.43 μmol/L in those with elevated IOP (*p* = 0.4, 95% CI 1.1–1.8). Mean Hcy levels in subjects with glaucoma were 11.2 ± 3.5 μmol/L compared to 11.7 ± 5.5 μmol/L in subjects without glaucoma and with normal IOP ≤ 21 mmHg (*p* = 0.4, 95% CI 1.2–2.1).	[27]
Hcy	POAG, NTG	*n* = 48 POAG, *n* = 15 NTG, *n* = 75 control/serum	Hcy levels were significantly higher (*p* = 0.002) in the POAG group compared to the NTG and control groups.	[28]
L-cysteine (Cys)	POAG, NTG, OHT	*n* = 44 POAG, *n* = 20 NTG, *n* = 52 OHT, *n* = 78 controls/plasma	Increased levels of Cys may be associated with glaucoma, especially in patients with POAG. The Cys levels in POAG and NTG groups were significantly higher (*p* = 0.007–0.043).	[25]
Endothelin-1 (ET-1)	POAG, NTG	*n* = 48 POAG, *n* = 15 NTG, *n* = 75 control/serum	The ET-1 levels were significantly higher (*p* = 0.002) in the POAG group compared to the NTG and control groups.	[28]
Brain-derived neurotrophic factor (BDNF)	POAG	*n* = 45 POAG, *n* = 15 control/serum	Serum levels of BDNF in glaucoma patients were significantly lower than those measured in healthy control patient (261.2 ± 75.0 pg/mL vs. 313.6 ± 79.6 pg/mL, *p* = 0.03). Subgroup analysis showed that serum levels of BDNF patients were significantly lower in early (253.8 ± 40.7 pg/mL, *p* = 0.019) and moderate glaucoma (231.3 ± 54.3 pg/mL, *p* = 0.04) but not in advanced glaucoma (296.2 ± 103.1 pg/mL, *p* = 0.06) compared to healthy control subjects.	[29]
Nerve growth factor (NGF)	POAG	*n* = 45 POAG, *n* = 15 control/serum	Serum levels of NGF in glaucoma patients were significantly lower than those measured in healthy control subject (4.1 ± 1 pg/mL vs. 5.5 ± 1.2 pg/mL, *p* = 0.01). Subgroup analysis showed that serum levels of NGF were significantly lower in early (3.5 ± 0.9 pg/mL, *p* = 0.0008) and moderate glaucoma (3.8 ± 0.7 pg/mL *p* < 0.0001) but not in advanced glaucoma (5.0 ± 0.7 pg/mL, *p* = 0.32) compared to healthy control subjects.	[29]
BDNF	NTG	*n* = 20 NTG, *n* = 20 control/tear	The mean level of BDNF detected in the tears of the normal subjects was 77.09 ± 4.84 ng/mL, and the BDNF level in the tears of case group subjects was 24.33 ± 1.48 ng/mL (*p* < 0.001).	[30]
BDNF	POAG	*n* = 25 POAG, *n* = 25 control/serum	Mean BDNF level in the serum was 27.16 ± 5.53 ng/mL in the control subjects and 18.42 ± 4.05 ng/mL in the subjects with early-stage glaucoma; there were no significant differences in serum BDNF levels according to the subjects’ age, gender, duration of glaucoma, mean IOP, or blood pressure (*p* > 0.05).	[31]
N-terminal fragment of the proatrial natriuretic peptide (NT-proANP, 1-98)	Glaucoma and cataract	*n* = 58 POAG, *n* = 32 cataract (control)/plasma and aqueous humor	The plasma NT-proANP concentration was significantly increased in patients with POAG compared to those in the control group (7.00 vs. 4.65 nmol/L, *p* = 0.0054). The NT-proANP concentration in the aqueous humor was significantly higher in the POAG patients (0.47 vs. 0.09 nmol/L, *p* = 0.0112). There was no correlation between the NT-proANP values in the aqueous humor and the plasma of the POAG patients or between the NT-proANP values in the aqueous humor and IOP.	[32]
Asymmetric dimethylarginine (ADMA), a dimethylated isomeric derivative of the amino acid l-arginine	Glaucoma	*n* = 211 glaucoma, *n* = 295 control/serum	A significant increase in serum ADMA concentration was detected in advanced glaucoma cases compared with control cases (*p* ≤ 0.0001).	[33]
Symmetric dimethylarginine (SDMA), a dimethylated isomeric derivative of the amino acid l-arginine	Glaucoma	*n* = 211 glaucoma, *n* = 295 control/serum	A significant increase in serum SDMA concentration was detected in advanced glaucoma cases compared with control cases (*p* ≤ 0.0001).	[33]
Ferritin	Glaucoma	*n* = 164,029/serum	The mean serum ferritin level was 56.98 ng/mL in women and 223.82 ng/mL in men.	[34]
Ferritin	Glaucoma	*n* = 17,476/serum	Participants whose serum ferritin level was greater than 61 ng/mL had significantly higher odds of a glaucoma diagnosis when compared with those with a level less than 31 ng/mL, after adjustment for potential confounders (ferritin levels of 31−61 ng/mL: odds ratio [OR], 1.17; 95% CI, 0.84−1.62; ferritin levels of 62−112 ng/mL: OR, 1.60; 95% CI, 1.16−2.20; and ferritin levels of 113−3018 ng/mL: OR, 1.89; 95% CI, 1.32−2.72).	[35]
Panel of 17 most differentially altered proteins	POAG, PEXG	*n* = 73(POAG), *n* = 59 (PEXG), *n* = 70 healthy controls/serum	Seventeen most differentially altered proteins identified in this analysis confirmed that they were also overexpressed in the intact serum of newly recruited glaucoma patients.	[36]
Main matricellular proteins (SPARC, thrombospondin-2, and osteopontin)	Acute primary angle closure (APAC), non-glaucomatous cataract	*n* = 29 APAC, *n* = 12 previous APAC, *n*= 22 cataract/aqueous humor	The levels of SPARC, thrombospondin-2, and osteopontin were significantly elevated in the APAC group as compared to the cataract group (*p* < 0.001, *p* < 0.001, and *p* = 0.009, respectively).	[37]

**Table 2:** Autoantibodies and antibodies evaluated as potential biomarkers in glaucoma.

Biomarker	Patient Diagnosis	Number of Patients (*n*)/Biological Material	Results	References
IgG	Primary open-angle glaucoma (POAG)	*n* = 111 POAG, *n* = 49 controls/serum	A number of serum proteins were identified and serve as candidate biomarkers of glaucoma. It is unclear whether the IgG-bound serum proteins identified in this study reflect disease-causing antigens.	[23]
IgG (heat shock protein 27, α-enolase, actin, and GAPDH)	POAG, pseudoexfoliation glaucoma (PEX)	*n* = 15 POAG, *n* = 14 PEX, *n* = 15 controls/aqueous humor	Significant differences were found between the IgG antibody profiles of the glaucoma groups (PEX and POAG) and the control subjects.	[51]
IgG	POAG, normal-tension glaucoma (NTG)	*n* = 19 POAG, *n* = 17 NTG, *n* = 30 controls/serum	All patients showed a complex repertoire of IgG antibodies against retinal, optic nerve, and optic nerve head antigens. The POAG group had the most significant difference against retinal antigens (*p* = 0.0021) compared with other antigens. In the NTG group, the highest reactivity appeared against the optic nerve head (*p* = 0.00053) and the optic nerve (*p* = 0.0025).	[50]
Autoantibody, IgG	POAG	*n* = 13 POAG, *n* = 15 controls/serum	In total, 75 peptides of the variable IgG domain showed significant glaucoma-related level changes (*p* < 0.05; log2 fold change ≥ 0.5): 6 peptides were highly abundant in POAG sera, whereas 69 peptides were minimally abundant in comparison to the control group.	[53]
Antibodies against trabecular meshwork	POAG	*n* = 60 POAG, *n* = 40 controls/serum	VDAC2, PGAM1, and CALD1, new autoantibodies, have been identified in association with POAG.	[49]
IgG antibodies against retinal antigens	NTG	*n* = 21 NTG, *n* = 21 controls/aqueous humor	α B-crystallin, vimentin, and heat-shock protein were analyzed as the antigen bands.	[52]

**Table 3:** Cytokines and growth factors evaluated as potential biomarkers in glaucoma.

Biomarker	Patient Diagnosis	Number of Patients (*n*)/Biological Material	Results	References
Interleukin-8 (IL-8) and 17-β-estradiol (E2)	Primary angle closure glaucoma (PACG) and processes related to menopause	*n* = 200 postmenopausal women with PACG, *n* = 151 healthy postmenopausal women (control)/serum	Decreased E2 (*p* = 0.007) and increased IL-8 (*p* < 0.001) levels were risk factors in postmenopausal women with PACG. A significant negative correlation was observed between IL-8 levels and E2 (*p* = 0.02).	[25]
TNF-α	Primary open-angle glaucoma (POAG)	*n* = 51 POAG patients *n* = 88 controls/plasma	The mean TNF-α level was significantly more increased in POAG cases than in control cases (*p* = 0.003). Logistic regression analysis showed that the risk of POAG was most significantly affected by TNF-α level (not by age and sex).	[57]
TNF-α	Pseudoexfoliation glaucoma (PEG)	*n* = 49 PEG patients *n* = 88 controls (non-glaucomatous patients)/plasma	The mean TNF-α concentration was significantly higher in PEG patients than in the control subjects (*p* = 0.000). A positive and significant correlation was observed between TNF-α concentration and cup/disc ratio, providing an important clinical index for PEG.	[58]
TNF-α, TNF receptors 1 (TNFR1) and 2 (TNFR2)	Boston keratoprosthesis patients with glaucoma (KPro G), Boston keratoprosthesis patients without glaucoma (KPro NoG), primary angle-closure glaucoma patients without Boston keratoprosthesis (PACG)	*n* = 19 KPro patients with glaucoma (KPro G), *n* = 12 KPro patients without glaucoma (KPro NoG), *n* = 13 patients with primary angle-closure glaucoma without KPro (PACG), *n* = 21 patients with narrow-angle without glaucoma or KPro (NA)/plasma	KPro G and KPro NoG patients had higher blood plasma levels of TNF-α (*p* = 0.006, and *p* = 0.04, respectively) compared to NA patients. KPro G patients had higher blood plasma levels of TNFR2 than NA patients (*p* = 0.048). KPro status was positively associated with TNF-α levels (*p* = 0.002) and TNFR2 levels (*p* = 0.035) after adjusting for age, gender, BMI, glaucoma status, and erythrocyte sedimentation rate (ESR).	[56]
Proinflammatory cytokines (IFNγ, IL-10, IL-12p70, IL-13, IL-1β, IL-2, IL-4, IL-6, IL-8, and TNFα)	POAG	*n* = 10 patients with early POAG, *n* = 9 control/human tear samples	Mean concentrations of tear-film cytokines were lower in the POAG group for 8 of 10 cytokines tested (for IFNγ, IL-12p70, IL-13, IL-1β, IL-2, IL-4, IL-6, IL-8). IL-12p70 was significantly lower in the tear films of patients with newly diagnosed POAG compared to control subjects (*p* = 0.035).	[55]
Brain-derived neurotrophic factor (BDNF), nerve growth factor (NGF)	POAG with a wide spectrum of disease severity	*n* = 45 patients affected by glaucoma at different stages, *n* = 15 control (age-matched)/serum	Serum levels of BDNF in glaucoma patients were significantly lower compared to healthy control subjects (*p* = 0.03). Additionally, serum levels of BDNF were significantly lower in early (*p* = 0.019) and moderate (*p* = 0.04) glaucoma but not in advanced glaucoma (*p* = 0.06) comparied to control subjects. Serum levels of NGF in glaucoma patients were significantly lower compared to control subjects (*p* = 0.01). Additonally, serum levels of NGF were significantly lower in early (*p* = 0.0008) and moderate glaucoma (*p* < 0.0001) but not in advanced glaucoma (*p* = 0.32) compared to healthy control subjects.	[29]
Insulin-like growth factor-1 (IGF-1)	Pseudoexfoliation (PEX) with or without glaucoma	*n* = 110 participants (age 65 years or older) who were divided into groups: 1. patients with PEX syndrome (*n* = 35), 2. patients with PEX glaucoma (*n* = 34), 3. participants without PEX or glaucoma (*n* = 41)/serum	Statistically significant differences between the groups in terms of IGF-1 concentration were not observed (*p* = 0.276). IGF-1 levels in circulation did not differ in the presence of PEX syndrome with or without glaucoma.	[63]
Transforming growth factor-β2 (TGFβ2), secreted frizzled-related protein-1 (SFRP1)	Different types of glaucoma	*n* = 105 patients divided into five groups: cataract (control), POAG, chronic angle-closure glaucoma (CACG), primary angle-closure suspects (PACS), and acute angle-closure glaucoma (AACG)/aqueous humor	The concentration of TGFβ2 in POAG patients (but not CACG, PACS, or AACG patients) was significantly higher compared to control subjectsDifferences in TGFβ2 concentration within AACG patients were observed after consideration of IOP (high and normal). The concentration of SFRP1 was not significantly different among the groups, but a statistically significant negative correlation between SFRP1 and IOP existed in the POAG group.	[66]

**Table 4:** Hormones and enzymes evaluated as potential biomarkers in glaucoma.

Hormone	Patient Diagnosis	Number of Patients (*n*)/Material	Results	References
Adrenocorticotropic hormone (ACTH)	RNFL thickness	*n* = 863/serum	Higher levels of ACTH were associated with thinner RNFL globally (*p* = 0.009). Lower adrenal sensitivity was associated with thinner RNFL inferotemporally (*p* < 0.001) and temporally (*p* = 0.037).	[68]
Cortisol	RNFL thickness	*n* = 863/serum	RNFL thickness was not associated with plasma cortisol levels.	[68]
Postmenopausal hormones	Primary open-angle glaucoma (POAG)	152,163/statistical analysis. Postmenopausal hormone medications containing estrogen only, estrogen + progesterone, and estrogen + androgen, as captured from outpatient pharmacy claims over a 4-year period	Of 152,163 eligible enrollees, 2925 (1.9%) developed POAG. After adjustment for confounding factors, each additional month of use of PMH containing estrogen only was associated with a 0.4% reduced risk for POAG (HR, 0.996 (95% CI, 0.993–0.999); *p* = 0.02). The risk for POAG did not differ with each additional month of use of estrogen + progesterone (HR, 0.994 (95% CI, 0.987–1.001); *p* = 0.08) or estrogen + androgen (HR, 0.999 (95% CI, 0.988–1.011); *p* = 0.89).	[67]
Superoxide dismutase (SOD), total antioxidant status (TAS), hydrogen peroxide (H_2_O_2_), malondialdehyde (MDA), glutathione peroxidase, glutathione reductase	Primary angle-closure glaucoma (PACG).	*n* = 94 PACG, *n* = 89 controls/serum	Serum levels of TAS, SOD, MDA, and H_2_O_2_ were independent risk/protective factors for PACG.	[70]
Superoxide dismutase 1 (SOD1)	POAG	*n* = 15 POAG, *n* = 11 controls/peripheral blood	The mRNA expression level of SOD1 showed significant downregulation in patients diagnosed with POAG.	[69]

**Table 5:** Uric acid as a potential biomarker in glaucoma.

Patient Diagnosis	Number of Patients (*n*)/Biological Material	Results	References
Primary open-angle glaucoma (POAG)	*n* = 163 POAG, *n* = 103 controls/serum	The level of serum UA in the POAG group (0.321 ± 0.084 mmol/L) was approximately 12.77% lower (*p* < 0.001) than that of the control group (0.362 ± 0.053 mmol/L). The UA/creatinine (Cr) ratio was approximately 14.99% lower (*p* < 0.001) in patients with POAG (4.47 ± 1.15) compared with the control group (5.14 ± 1.05).	[72]
Primary angle closure glaucoma (PACG)	*n* = 886 PACG, *n* = 994 control/serum	The levels of UA were significantly lower (*p* = 0.025) in PACG patients (0.286 ± 0.082 mmol/L) compared with control subjects (0.295 ± 0.085 mmol/L). The mean serum UA levels were lowest in the severe group (0.281 ± 0.074 mmol/L) followed by moderate (0.282 ± 0.080 mmol/L) and mild (0.297 ± 0.090 mmol/L), with significant differences among the three groups (*p* = 0.032).	[73]

## References

[1] Tuulonen A., Airaksinen P.J., Erola E., Forsman E., Friberg K., Kaila M., Klemetti A., Mäkelä M., Oskala P., Puska P. (2003). The Finnish evidence-based guideline for open angle glaucoma. Acta Ophthalmol. Scand..

[2] Shon K., Wollstein G., Schuman J.S., Sung K.R. (2014). Prediction of glaucomatous field progression: Pointwise analysis. Curr. Eye Res..

[3] Weinreb R.N., Khaw P.T. (2004). Primary open-angle glaucoma. Lancet.

[4] Rudnicka A.R., Mt-Isa S., Owen C.G., Cook D.G., Ashby D. (2006). Variations in primary open-angle glaucoma prevalence by age, gender, and race: A Bayesian meta-analysis. Invest Ophthalmol. Vis. Sci..

[5] EGS Foundation (2017). European Glaucoma Society Terminology and Guidelines for Glaucoma, 4th Edition—Part 1. Supported by the EGS Foundation. Br. J. Ophthalmol..

[6] Nakabayashi M. (2004). Review of the ischemia hypothesis for ocular hypertension other than congenital glaucoma and closed-angle glaucoma. Ophthalmologica.

[7] Tezel G. (2011). The immune response in glaucoma: A perspective on the roles of oxidative stress. Exp. Eye Res..

[8] Shazly T.A., Aljajeh M., Latina M.A. (2011). Autoimmune basis of glaucoma. Semin. Ophthalmol..

[9] Tezel G. (2013). A proteomics view of the molecular mechanisms and biomarkers of glaucomatous neurodegeneration. Prog. Retin. Eye Res..

[10] Soto I., Howell G.R. (2014). The complex role of neuroinflammation in glaucoma. Cold Spring Harb. Perspect. Med..

[11] Chidlow G., Wood J.P.M., Casson R.J. (2017). Investigations into hypoxia and oxidative stress at the optic nerve head in a rat model of glaucoma. Front. Neurosci..

[12] Quigley H.A., Broman A.T. (2006). The number of people with glaucoma worldwide in 2010 and 2020. Br. J. Ophthalmol..

[13] Tezel G., Yang X., Luo C., Kain A.D., Powell D.W., Kuehn M.H., Kaplan K.J. (2010). Oxidative stress and the regulation of complement activation in human glaucoma. Invest. Ophthalmol. Vis. Sci..

[14] Yanagi M., Kawasaki R., Wang J.J., Wong T.Y., Crowston J., Kiuchi Y. (2011). Vascular risk factors in glaucoma: A review. Clin. Exp. Ophthalmol..

[15] Bell K., Gramlich O.W., Von Thun Und Hohenstein-Blaul N., Beck S., Funke S., Wilding C., Pfeiffer N., Grus F.H. (2013). Does autoimmunity play a part in the pathogenesis of glaucoma?. Prog. Retin. Eye Res..

[16] Grzybowski A., Och M., Kanclerz P., Leffler C., De Moraes C.G. (2020). Primary open angle glaucoma and vascular risk factors: A review of population based studies from 1990 to 2019. J. Clin. Med..

[17] McMonnies C.W. (2017). Glaucoma history and risk factors. J. Optom..

[18] Hohenstein-Blaul N.V.T.U., Kunst S., Pfeiffer N., Grus F.H. (2017). Biomarkers for glaucoma: From the lab to the clinic. Eye.

[19] Hondur G., Göktas E., Yang X., Al-Aswad L., Auran J.D., Blumberg D.M., Cioffi G.A., Liebmann J.M., Suh L.H., Trief D. (2017). Oxidative stress-related molecular biomarker candidates for glaucoma. Investig. Ophthalmol. Vis. Sci..

[20] Beykin G., Goldberg J.L. (2019). Molecular biomarkers for glaucoma. Curr. Ophthalmol. Rep..

[21] Biomarkers Definitions Working Group (2001). Biomarkers and surrogate endpoints: Preferred definitions and conceptual framework. Clin. Pharmacol. Ther..

[22] Strimbu K., Tavel J.A. (2010). What are biomarkers?. Curr. Opin. HIV AIDS.

[23] Tezel G., Thornton I.L., Tong M.G., Luo C., Yang X., Cai J., Powell D.W., Soltau J.B., Liebmann J.M., Ritch R. (2012). Immunoproteomic analysis of potential serum biomarker candidates in human glaucoma. Invest. Ophthalmol. Vis. Sci..

[24] Damodaran S., Damodaran S., Parkin K.L. (2008). Amino Acids, Peptides, and Proteins. Fennema’s Food Chemistry.

[25] Lin Z., Huang S., Yu H., Sun J., Huang P., Zhong Y. (2020). Analysis of plasma hydrogen sulfide, homocysteine, and L-cysteine in open-angle flaucoma patients. J. Ocul. Pharmacol. Ther..

[26] Lee W.J., Na K.I., Kim Y.K., Jeoung J.W., Park K.H. (2017). Diagnostic ability of wide-field retinal nerve fiber layer maps using swept-source optical coherence tomography for detection of preperimetric and early perimetric glaucoma. J. Glaucoma.

[27] Leibovitzh H., Cohen E., Levi A., Kramer M., Shochat T., Goldberg E., Krause I. (2016). Relationship between homocysteine and intraocular pressure in men and women: A population-based study. Medicine.

[28] López-Riquelme N., Villalba C., Tormo C., Belmonte A., Fernandez C., Torralba G., Hernández F. (2015). Endothelin-1 levels and biomarkers of oxidative stress in glaucoma patients. Int. Ophthalmol..

[29] Oddone F., Roberti G., Micera A., Busanello A., Bonini S., Quaranta L., Agnifili L., Manni G. (2017). Exploring serum levels of brain derived neurotrophic factor and nerve frowth factor across glaucoma stages. PLoS ONE.

[30] Ghaffariyeh A., Honarpisheh N., Heidari M.H., Puyan S., Abasov F. (2011). Brain-derived neurotrophic factor as a biomarker in primary open-angle glaucoma. Optom. Vis. Sci..

[31] Ghaffariyeh A., Honarpisheh N., Shakiba Y., Puyan S., Chamacham T., Zahedi F., Zarrineghbal M. (2009). Brain-derived neurotrophic factor in patients with normal-tension glaucoma. Optometry.

[32] Baumane K., Ranka R., Laganovska G. (2017). Association of NT-proANP level in plasma and humor aqueous with primary open-angle glaucoma. Curr. Eye Res..

[33] Javadiyan S., Burdon K.P., Whiting M.J., Abhary S., Straga T., Hewitt A.W., Mills R.A., Craig J.E. (2012). Elevation of serum asymmetrical and symmetrical dimethylarginine in patients with advanced glaucoma. Investig. Ophthalmol. Vis. Sci..

[34] Gye H.J., Kim J.M., Yoo C., Shim S.H., Won Y.S., Sung K.C., Lee M.Y., Park K.H. (2016). Relationship between high serum ferritin level and glaucoma in a South Korean population: The Kangbuk Samsung health study. Br. J. Ophthalmol..

[35] Lin S.C., Wang S.Y., Yoo C., Singh K., Lin S.C. (2014). Association between serum ferritin and glaucoma in the South Korean population. JAMA Ophthalmol..

[36] González-Iglesias H., Álvarez L., García M., Escribano J., Rodríguez-Calvo P.P., Fernández-Vega L., Coca-Prados M. (2014). Comparative proteomic study in serum of patients with primary open-angle glaucoma and pseudoexfoliation glaucoma. J. Proteom..

[37] Wang J., Fu M., Liu K., Wang N., Zhang Z., Zhou M., Xu X. (2018). Matricellular proteins play a potential role in acute primary angle closure. Curr. Eye Res..

[38] Farkas R.H., Chowers I., Hackam A.S., Kageyama M., Nickells R.W., Otteson D.C., Duh E.J., Wang C., Valenta D.F., Gunatilaka T.L. (2004). Increased expression of iron-regulating genes in monkey and human glaucoma. Invest. Ophthalmol. Vis. Sci..

[39] Wiesmann C., de Vos A.M. (2001). Nerve growth factor: Structure and function. Cell Mol Life Sci..

[40] Binder D.K., Scharfman H.E. (2004). Brain-derived neurotrophic factor. Growth Factors.

[41] Skaper S.D. (2012). The neurotrophin family of neurotrophic factors: An overview. Methods Mol. Biol..

[42] Wu G. (2013). Amino Acids: Biochemistry and Nutrition.

[43] Blom H.J., Smulders Y. (2011). Overview of homocysteine and folate metabolism. With special references to cardiovascular disease and neural tube defects. J. Inherit. Metab. Dis..

[44] Ye S., Chang Y., Kim C.W., Kwon M.J., Choi Y., Ahn J., Kim J.M., Kim H.S., Shin H., Ryu S. (2015). Intraocular pressure and coronary artery calcification in asymptomatic men and women. Br. J. Ophthalmol..

[45] Stow L.R., Jacobs M.E., Wingo C.S., Cain B.D. (2011). Endothelin-1 gene regulation. FASEB J..

[46] Ludwig R.J., Vanhoorelbeke K., Leypoldt F., Kaya Z., Bieber K., McLachlan S.M., Komorowski L., Luo J., Cabral-Marques O., Hammers C.M. (2017). Mechanisms of autoantibody-induced pathology. Front. Immunol..

[47] Grus F.H., Joachim S.C., Bruns K., Lackner K.J., Pfeiffer N., Wax M.B. (2006). Serum autoantibodies to alpha-fodrin are present in glaucoma patients from Germany and the United States. Invest. Ophthalmol. Vis. Sci..

[48] Gramlich O.W., Bell K., von Thun Und Hohenstein-Blaul N., Wilding C., Beck S., Pfeiffer N., Grus F.H. (2013). Autoimmune biomarkers in glaucoma patients. Curr. Opin. Pharmacol..

[49] Beutgen V.M., Perumal N., Pfeiffer N., Grus F.H. (2019). Autoantibody biomarker discovery in primary open angle glaucoma using serological proteome analysis (SERPA). Front. Immunol..

[50] Joachim S.C., Pfeiffer N., Grus F.H. (2005). Autoantibodies in patients with glaucoma: A comparison of IgG serum antibodies against retinal, optic nerve, and optic nerve head antigens. Graefes Arch. Clin. Exp. Ophthalmol..

[51] Joachim S.C., Wuenschig D., Pfeiffer N., Grus F.H. (2007). IgG antibody patterns in aqueous humor of patients with primary open angle glaucoma and pseudoexfoliation glaucoma. Mol. Vis..

[52] Joachim S.C., Bruns K., Lackner K.J., Pfeiffer N., Grus F.H. (2007). Antibodies to alpha B-crystallin, vimentin, and heat shock protein 70 in aqueous humor of patients with normal tension glaucoma and IgG antibody patterns against retinal antigen in aqueous humor. Curr. Eye Res..

[53] Schmelter C., Perumal N., Funke S., Bell K., Pfeiffer N., Grus F.H. (2017). Peptides of the variable IgG domain as potential biomarker candidates in primary open-angle glaucoma (POAG). Hum. Mol. Genet..

[54] Li S., Zhang H., Shao M., Li Y., Song Y., Sun X., Cao W. (2020). Association between 17-β-estradiol and interleukin-8 and visual field progression in postmenopausal omen with primary angle closure glaucoma. Am. J. Ophthalmol..

[55] Gupta D., Wen J.C., Huebner J.L., Stinnett S., Kraus V.B., Tseng H.C., Walsh M. (2017). Cytokine biomarkers in tear film for primary open-angle glaucoma. Clin. Ophthalmol..

[56] Paschalis E.I., Taniguchi E.V., Chodosh J., Pasquale L.R., Colby K., Dohlman C.H., Shen L.Q. (2019). Blood levels of tumor necrosis factor alpha and its type 2 receptor are elevated in patients with Boston Type I Keratoprosthesis. Curr. Eye Res..

[57] Kondkar A.A., Sultan T., Almobarak F.A., Kalantan H., Al-Obeidan S.A., Abu-Amero K.K. (2018). Association of increased levels of plasma tumor necrosis factor alpha with primary open-angle glaucoma. Clin. Ophthalmol..

[58] Kondkar A.A., Azad T.A., Almobarak F.A., Kalantan H., Al-Obeidan S.A., Abu-Amero K.K. (2018). Elevated levels of plasma tumor necrosis factor alpha in patients with pseudoexfoliation glaucoma. Clin. Ophthalmol..

[59] Mohammadi A., Amooeian V.G., Rashidi E. (2018). Dysfunction in brain-derived neurotrophic factor signaling pathway and susceptibility to schizophrenia, Parkinson’s and Alzheimer’s diseases. Curr. Gene Ther..

[60] Arvat E., Broglio F., Ghigo E. (2000). Insulin-like growth factor I: Implications in aging. Drugs Aging.

[61] Puche J.E., Castilla-Cortázar I. (2012). Human conditions of insulin-like growth factor-I (IGF-I) deficiency. J. Transl. Med..

[62] Ferreira S.T. (2021). Brain insulin, insulin-like growth factor 1 and glucagon-like peptide 1 signalling in Alzheimer’s disease. J. Neuroendocrinol..

[63] Dogan A.S., Kabatas N., Erden G., Celikay O., Arzuhal A.E., Gurdal C. (2017). Serum insulin-like growth factor-1 levels in patients with pseudoexfoliation syndrome and glaucoma. Int. Ophthalmol..

[64] Schlötzer-Schrehardt U., Naumann G.O. (2006). Ocular and systemic pseudoexfoliation syndrome. Am. J. Ophthalmol..

[65] Ekström C., Kilander L. (2014). Pseudoexfoliation and Alzheimer’s disease: A population-based 30-year follow-up study. Acta Ophthalmol..

[66] Guo T., Guo L., Fan Y., Fang L., Wei J., Tan Y., Chen Y., Fan X. (2019). Aqueous humor levels of TGFβ2 and SFRP1 in different types of glaucoma. BMC Ophthalmol..

[67] Newman-Casey P.A., Talwar N., Nan B., Musch D.C., Pasquale L.R., Stein J.D. (2014). The potential association between postmenopausal hormone use and primary open-angle glaucoma. JAMA Ophthalmol..

[68] Lee S.S., Sanfilippo P.G., Yazar S., Pennell C.E., Hewitt A.W., Wang C.A., Martin W.N., Mackey D.A. (2020). Do levels of stress markers influence the retinal nerve fiber layer thickness in young adults?. J. Glaucoma.

[69] Canizales L., Rodriguez L., Rivera C., Martinez A., Mendez F., Castillo A. (2016). Low-level expression of SOD1 in peripheral blood samples of patients diagnosed with primary open-angle glaucoma. Biomark. Med..

[70] Li S., Shao M., Li Y., Li X., Wan Y., Sun X., Cao W. (2020). Relationship between oxidative stress biomarkers and visual field progression in patients with primary angle closure glaucoma. Oxid. Med. Cell Longev..

[71] Glantzounis G.K., Tsimoyiannis E.C., Kappas A.M., Galaris D.A. (2005). Uric acid and oxidative stress. Curr. Pharm. Des..

[72] Li S., Shao M., Li D., Tang B., Cao W., Sun X. (2019). Association of serum uric acid levels with primary open-angle glaucoma: A 5-year case-control study. Acta Ophthalmol..

[73] Li S., Shao M., Tang B., Zhang A., Cao W., Sun X. (2017). The association between serum uric acid and glaucoma severity in primary angle closure glaucoma: A retrospective case-control study. Oncotarget.

[74] Good D.M., Thongboonkerd V., Novak J., Bascands J.L., Schanstra J.P., Coon J.J., Dominiczak A., Mischak H. (2007). Body fluid proteomics for biomarker discovery: Lessons from the past hold the key to success in the future. J. Proteom. Res..

[75] Thongboonkerd V. (2007). Proteomics of Human Body Fluids. Principles, Methods, and Applications.

[76] Zhou L., Beuerman R.W., Foo Y., Liu S., Ang L.P., Tan D.T. (2006). Characterisation of human tear proteins using high-resolution mass spectrometry. Ann. Acad. Med. Singap..

[77] Zhou L., Zhao S.Z., Koh S.K., Chen L., Vaz C., Tanavde V., Li X.R., Beuerman R.W. (2012). In-depth analysis of the human tear proteome. J. Proteom..

[78] Pan C.W., Ke C., Chen Q., Tao Y.J., Zha X., Zhang Y.P., Zhong H. (2020). Differential metabolic markers associated with primary open-angle glaucoma and cataract in human aqueous humor. BMC Ophthalmol..

[79] Celis J.E., Gromov P. (1999). 2D protein electrophoresis: Can it be perfected?. Curr. Opin. Biotechnol..

[80] Boehm N., Wolters D., Thiel U., Lossbrand U., Wiegel N., Pfeiffer N., Grus F.H. (2012). New insights into autoantibody profiles from immune privileged sites in the eye: A glaucoma study. Brain Behav. Immun..

[81] Hosseini S., Vázquez-Villegas P., Rito-Palomares M., Martinez-Chapa S.O., Hosseini S., Vázquez-Villegas P., Rito-Palomares M., Martinez-Chapa S.O. (2018). Advantages, disadvantages and modifications of conventional ELISA. Enzyme-Linked Immunosorbent Assay (ELISA).

[82] Aretz I., Meierhofer D. (2016). Advantages and pitfalls of mass spectrometry based metabolome profiling in systems biology. Int. J. Mol. Sci..

